# Dickdarmerkrankungen in Computertomographie und Magnetresonanztomographie

**DOI:** 10.1007/s00117-023-01150-7

**Published:** 2023-05-23

**Authors:** Martina Scharitzer, Katharina Lampichler, Sabine Popp, Thomas Mang

**Affiliations:** Universitätsklinik für Radiologie und Nuklearmedizin, Medizinische Universität Wien, Waehringer Guertel 18–20, 1090 Wien, Österreich

**Keywords:** Kolonerkrankungen, Kolitis, Inflammation, Neoplasie, CT-Kolonographie, Colonic diseases, Colitis, Inflammation, Neoplastic processes, CT colonography

## Abstract

**Hintergrund:**

Eine frühzeitige Diagnose von luminalen Dickdarmerkrankungen ist von wesentlicher klinischer Bedeutung, um eine rechtzeitige optimierte Therapie beginnen und Komplikationen frühzeitig erkennen zu können.

**Ziel der Arbeit:**

Diese Arbeit soll einen Überblick über den Einsatz radiologischer Methoden bei der Diagnose neoplastischer und entzündlicher luminaler Erkrankungen des Kolons vermitteln. Dabei werden charakteristische morphologische Merkmale diskutiert und gegenübergestellt.

**Material und Methoden:**

Anhand einer ausführlichen Literaturrecherche wird der aktuelle Wissensstand bezüglich der bildgebenden Diagnostik luminaler Pathologien des Dickdarms und ihrer Bedeutung im Patientenmanagement dargestellt.

**Ergebnisse:**

Durch die technologischen Fortschritte in der Bildgebung ist die Diagnose von neoplastischen und entzündlichen Kolonerkrankungen mittels abdominaler Computertomographie (CT) und Magnetresonanztomographie (MRT) zum etablierten Standard geworden. Die Bildgebung erfolgt im Rahmen der Erstdiagnose bei klinisch symptomatischen Patienten, zum Ausschluss von Komplikationen, für eine Verlaufsbeurteilung unter Therapie sowie als optionale Screeningmethode bei asymptomatischen Personen.

**Diskussion:**

Die genaue Kenntnis der radiologischen Erscheinungsformen der zahlreichen luminalen Krankheitsbilder, dem typischen Verteilungsmuster und den charakteristischen Darmwandveränderungen sind wesentlich, um die diagnostische Entscheidungsfähigkeit zu verbessern.

## Abstract (en)

**Background:**

Early diagnosis of a luminal colonic disease is of essential clinical importance to start timely optimised therapy and detect complications early.

**Objectives:**

This paper aims to provide an overview of the use of radiological methods in diagnosing neoplastic and inflammatory luminal diseases of the colon. Characteristic morphological features are discussed and compared.

**Materials and methods:**

Based on an extensive literature review, the current state of knowledge regarding the imaging diagnosis of luminal pathologies of the colon and their importance in patient management is presented.

**Results:**

Technological advances in imaging have made the diagnosis of neoplastic and inflammatory colonic diseases using abdominal computed tomography and magnetic resonance imaging the established standard. Imaging is performed as part of the initial diagnosis in clinically symptomatic patients, to exclude complications, as a follow-up assessment under therapy and as an optional screening method in asymptomatic individuals.

**Conclusions:**

Accurate knowledge of the radiological manifestations of the numerous luminal disease patterns, the typical distribution pattern and characteristic bowel wall changes are essential to improve diagnostic decision-making.

Die radiologische Bildgebung des Kolons hat in den letzten Jahrzehnten durch technologische Fortschritte bedeutende Weiterentwicklungen erlebt und zu einem breiteren Einsatz der bildgebenden Diagnostik in der Abklärung von Kolonpathologien geführt. Obwohl es in der Diagnose entzündlicher Dickdarmerkrankungen viele überlappende radiologische Merkmale gibt, kann eine systemische Bildanalyse wichtige Anhaltspunkte liefern, um eine exakte Diagnosefindung zu erleichtern und in Zusammenschau mit endoskopischen Biopsien, Stuhlkulturen oder anderen klinischen Merkmalen das weitere Management wesentlich zu optimieren.

## Bildgebende Modalitäten

Die Computertomographie (CT) hat sich zur primären radiologischen Modalität für die Beurteilung von Pathologien des Kolons entwickelt. Besonders in der Akutdiagnostik stellt sie bei PatientInnen mit unspezifischen Bauchschmerzen oder bei Verdacht auf eine Obstruktion eine wichtige erste Diagnosemöglichkeit dar. Die genaue Evaluation der Darmwand, der Veränderungen des perikolischen Fettgewebes, des Mesenteriums sowie des Peritoneums ergibt wesentliche Anhaltspunkte zur spezifischen Diagnose kolorektaler Erkrankungen und ihrer Komplikationen. Die CT-Kolonographie als nichtinvasive Alternative zur Koloskopie erlaubt eine Beurteilung des Darmlumens und der perikolischen Strukturen und wird sowohl zur Symptomabklärung als auch zur Vorsorge eingesetzt.

Die Magnetresonanztomographie (MRT) ist eine vielversprechende Alternative zur CT bei der Abklärung akuter Bauchschmerzen ohne Anwendung ionisierender Strahlen. Bedeutende Fortschritte in der technischen Ausstattung der MR-Geräte ermöglichen eine schnelle Bildgebung des Darmtrakts mit hoher räumlicher Auflösung und ohne störende Bewegungsartefakte. Der große Weichteilkontrast ermöglicht eine hervorragende Darstellung von Darminfektionen, Entzündungen und Tumoren. Die wissenschaftliche Evidenz zum klinischen Einsatz der MR-Kolonographie ist allerdings begrenzt und die Untersuchung noch nicht ausreichend gut evaluiert. Die längere Untersuchungsdauer sowie auch die Notwendigkeit der Gabe von intravenösem Kontrastmittel sind Limitationen für die klinische Anwendung und den Einsatz als Screeningmethode. Die bisher publizierten Daten zeigen eine geringere Sensitivität für fortgeschrittene Neoplasien im Vergleich zur CT-Kolonographie [1].

Sowohl bei der CT als auch der MRT lässt sich eine luminale Pathologie des Kolons im nichtakuten Setting am besten bei guter Distension beurteilen. Neben einer primären rektalen Füllung ist auch eine längere Wartezeit nach oraler Füllung im Rahmen einer Enterographie geeignet, um pathologische Veränderungen des Dickdarms zu diagnostizieren [2]. Neutrale oder negative Kontrastmittel in Kombination mit intravenöser Kontrastmittelapplikation erlauben dabei eine optimale Abgrenzbarkeit von Pathologien des Dickdarms. Auf die Indikationen und Technik der CT-Kolonographie sowie die MRT des Rektums wird in anderen Übersichtsartikeln dieser Ausgabe näher eingegangen.

## Normale Anatomie

Die Breite der Kolonwand ist vom Ausmaß der intestinalen Distension abhängig. Bei guter Füllung ist die Wandbreite mit weniger als 3 mm sehr dünn. Bei schlechter Distension nimmt sie auf bis zu 3–5 mm zu und kann innerhalb einer spastischen Kontraktion bis zu 6–8 mm betragen [3]. Nach Kontrastmittelapplikation kann die Mukosa als eigene Wandschicht abgegrenzt werden, wohingegen die Submukosa als weniger vaskularisierte Schicht nur bei pathologischen Veränderungen wie Ödem, Einblutung oder Fetteinlagerung sichtbar wird. Das morphologische Erscheinungsbild, der Schweregrad, das Verteilungsmuster und das Fortschreiten luminaler und muraler Pathologien weisen oft auf eine spezifische Diagnose von Kolonerkrankungen hin.

## Neoplastische Veränderungen

### Polypen

**Figure 1:**
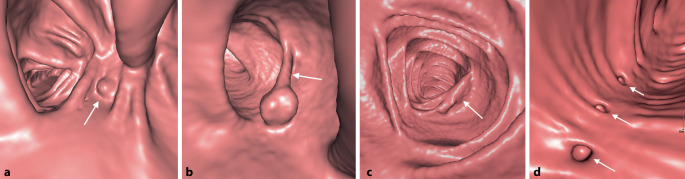

Zur Differenzierung luminaler kolorektaler Läsionen ist die genaue Beurteilung der Morphologie sowie der CT-Dichte einschließlich des Verhaltens nach Kontrastmittelapplikation entscheidend. Bei einer CT-Kolonographie wird darüber hinaus durch die Untersuchung in Rücken- und Bauchlage auch die Lagestabilität der Läsion beurteilt. Neben sessilen Polypen als runde oder lobulierte Füllungsdefekte (Abb. 1a) und gestielten Polypen mit einer länglichen Verbindung zur Darmwand (Abb. 1b) stellen insbesondere flache Läsionen eine radiologische Herausforderung dar. Diese oft unscheinbaren plaqueförmigen Wandverdickungen können schwer abgrenzbar sein (Abb. 1c), weisen aber oft einen pathognomonischen Kontrastmittelbelag („contrast coating“) auf. Eine Differenzierung zwischen den histologischen Subtypen sowie dem Vorliegen dysplastischer Anteile polypöser Veränderungen ist mittels radiologischer Bildgebung nicht möglich. Das wichtigste bildgebende Kriterium für die Bestimmung der klinischen Relevanz und des weiteren Managements von Kolonpolypen stellt die Größe dar [4].

Differenziert werden müssen adenomatöse Polypen von submukös lokalisierten Lipomen, die als gutartige Veränderung meist eine Größe von 1–3 cm aufweisen. Diese sind anhand der homogen fettäquivalenten Binnenstruktur und der typischerweise glatten Oberfläche leicht erkennbar.

Hereditäre gastrointestinale Polyposis-Syndrome sind eine Gruppe von Erkrankungen, die durch kongenitale genetische Mutationen verursacht werden und mit einem erhöhten Risiko für Darmneoplasien einhergehen. Sie sind für ca. 5–10 % der gastrointestinalen Krebsfälle verantwortlich [5]. Beim Lynch-Syndrom, dem hereditären kolorektalen Karzinom ohne Polyposis-Syndrom (HNPCC), finden sich Neoplasien in mehr als 70 % der Fälle im rechtsseitigen Kolon; diese entwickeln sich sehr rasch aus flachen Adenomen [5]. Die CT-Kolonographie ist zur Abklärung dieser Krankheitsbilder aufgrund der zwingenden Notwendigkeit des histologischen Neoplasieausschlusses und der einhergehenden Strahlenbelastung bei eher jüngeren PatientInnen nicht geeignet [6]. Seltenere Syndrome sind die familiär-adenomatöse Polypose, das juvenile Polyposis-Syndrom, das Peutz-Jeghers-Syndrom und das Cowden-Syndrom.

### Kolorektale Karzinome

Die kolorektalen Karzinome entwickeln sich in ca. 70–80 % der Fälle aus adenomatösen Polypen über einen Zeitraum von 10 bis 15 Jahren im Rahmen einer Mehrschritt-Karzinogenese, der sog. *Adenom-Karzinom-Sequenz*. Das Karzinomrisiko steigt mit der Polypengröße an. Es ist bei Adenomen < 1 cm sehr gering, liegt bei einer Größe zwischen 6 und 9 mm bei unter 1 % und ist bei Läsionen < 6 mm praktisch nicht nachweisbar [7]. Adenome > 1 cm, mit einem mehr als 25 %igen villösen Anteil sowie hochgradigen Dysplasien werden als fortgeschrittene Adenome bezeichnet und haben ein höheres Entartungsrisiko. Die übrigen kolorektalen Karzinome entstehen aus serratierten Adenomen über eine *serratierte Route* mit unterschiedlichen genetischen und epigenetischen Profilen [8].

Das kolorektale Karzinom stellt trotz jüngster Fortschritte bei therapeutischen und diagnostischen Verfahren aufgrund seiner hohen Inzidenz und Mortalität ein großes gesundheitspolitisches Problem dar. Es verursacht in Deutschland ca. 60.000 und in Österreich ca. 4500 Neuerkrankungen pro Jahr mit einer 5‑Jahres-Überlebensrate von 65 % [9, 10]. Bei einer generell geringen Abnahme der Gesamtinzidenz und der Mortalität von DarmkrebspatientInnen ist allerdings eine relative Zunahme bei jungen PatientInnen < 50 Jahre auffällig mit einer in den USA inzwischen am häufigsten diagnostizierten Krebsart bei Männern dieser Altersgruppe [11].

**Figure 2:**
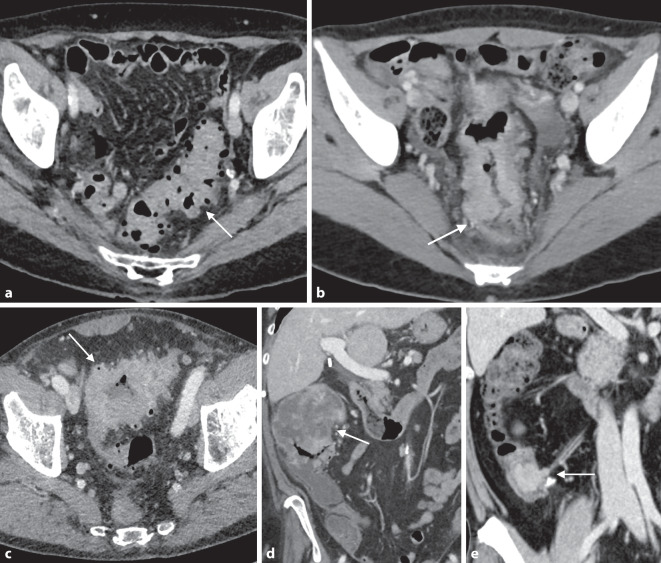

Radiomorphologisch ist ein kolorektales Karzinom charakterisiert durch eine eher kurzstreckige Wandverbreiterung mit einer polypoiden semizirkulär bis zirkulär stenotischen Konfiguration. Typisch ist ein abrupter Übergang von normaler Darmwand zur pathologischen Läsion, die im Randbereich eine überhängende Berandung aufweisen kann (*Schulterformation*; Abb. 2b). Zirkulär stenosierende Tumoren werden auch „apple core lesion“ genannt. Dickdarmneoplasien zeigen ein signifikantes Kontrastmittel-Enhancement, wobei zentral hypodense Areale auf eine nekrotische Einschmelzung bzw. Ulzerationen hinweisen. Etwa 10 % aller Kolonkarzinome bestehen histologisch zum überwiegenden Anteil aus Muzin mit unterschiedlichen klinisch-pathologischen Charakteristika, verschiedenen molekularen Eigenschaften und einem meist schlechteren Therapieansprechen. Bildgebende Hinweise auf ein muzinöses Kolonkarzinom sind ein heterogenerer Aufbau mit geringerer Dichte (Abb. 2d), eine stärkere und deutlicher exzentrische Wandverdickung sowie das Auftreten intraläsionaler Verkalkungen [12].

Die Rolle der präoperativen Bildgebung zur Beurteilung des lokalen Tumorstadiums wird noch untersucht, da die neoadjuvante Behandlung und Strahlentherapie die Überlebensrate im Vergleich zur operativen Resektion bisher nicht signifikant verbessert haben. Eine sichere Differenzierung zwischen einer rein mukosal/submukosalen Läsion (T1) und einer die Muscularis propria infiltrierenden Läsion (T2) ist derzeit bildgebend nicht möglich. Eine Infiltration der perikolischen Strukturen (T3) ist durch eine Verdichtung des angrenzenden Fettgewebes und eine unscharfe Begrenzung der Außenwand des Dickdarms mit zum Teil nodulären Tumorausläufern in das perikolische Fettgewebe erkennbar. Die CT weist in einer Metaanalyse eine gepoolte Sensitivität von 90 % für die Detektion einer Wandüberschreitung auf, mit einer Sensitivität von 77 % für die Abgrenzung einer perikolischen Invasionstiefe > 5 mm, entsprechend einem T‑Stadium > T3b. Die CT-Kolonographie zeigt dabei eine höhere Genauigkeit in der Differenzierung der Tumorstadien T1–T3ab von T3cd–T4 [13]. Aufgrund der begrenzten Rolle des lokoregionären Stagings liegt der primäre Fokus der präoperativen Bildgebung des Kolonkarzinoms daher auf der Beurteilung der Umgebungsinfiltration und dem Vorhandensein von Fernmetastasen [14].

Studien weisen darauf hin, dass die Dual-Energy-CT mit virtuell monochromatischen Bildern mit einer besseren Kontrastverstärkung im Vergleich zu polychromatisch standardmäßigen CT-Bildern das Tumorgrading mit der Detektion einer Mikrosatelliteninstabilität [15] oder der Erfassung von High-grade-Charakteristika verbessern kann [16]. Der Einsatz computerbasierter automatischer Erkennungssysteme hat eine erhöhte Sensitivität bei der Detektion von Darmpolypen gezeigt [17]. Auch die Anwendung maschinellen Lernens wird zunehmend erforscht, um mittels automatisierter Bildverarbeitungsmodalitäten sowie Segmentierungen und Bildfusionierungen die großen Datenmengen in der klinischen Praxis für eine optimierte Therapie zu nutzen [18].

### Seltene Neoplasien des Kolons

Seltenere Neoplasien des Dickdarms umfassen neuroendokrine Tumoren (NET), das Lymphom, lymphatische Neoplasien sowie Sarkome und Metastasen. Neuroendokrine Tumoren der Appendix werden meist im Rahmen einer Appendektomie diagnostiziert, wohingegen NET des Kolons meist größer sind. Sie können mit einer lokal-mesenterialen oder einer Fernmetastasierung einhergehen (Abb. 2e).

Lymphome weisen eine oft ausgedehnte fokale oder diffuse Wandverbreiterung auf, die jedoch meist nicht obstruktiv ist und typischerweise durch die Tumorinfiltration der Darmwand zu einer aneurysmatischen Erweiterung des Darmlumens mit fehlender prästenotischer Dilatation führt. Innerhalb des betroffenen Segments sind ein Verlust der Darmwandschichtung sowie eine nur geringe murale Kontrastmittelaufnahme auffällig (Abb. 2c). Sarkome können als kleinere Tumoren homogen solide erscheinen, bei einer zunehmenden Größe jedoch ausgedehnte zentrale Nekrosen aufweisen, die von einem Ring mit höheren Dichtewerten umgeben sind [19]. Gastrointestinale Stromatumoren sind im Kolon selten und abhängig von der Größe ebenfalls oft heterogen aufgrund von Nekrosen, Einblutungen, Ulzerationen oder zystischen Degenerationen.

### Divertikelkrankheit

Divertikel sind bei asymptomatischen PatientInnen der häufigste Befund des Kolons in der westlichen Welt. Sie sind als Ausstülpungen der Mukosa und Submukosa durch die Muskularis (Pseudodivertikel), selten als Herniationen der gesamten Darmwand (echte Divertikel) charakterisiert. Radiologisch ist eine Differenzierung zwischen beiden Typen nicht möglich. Am häufigsten sind das Colon sigmoideum und descendens betroffen mit einer Divertikelgröße von 2 mm bis zu 2 cm.

In der virtuell-endoskopischen 3D-Ansicht unterscheidet sich ein Divertikel von einem Polypen durch eine scharfe äußere Begrenzung und eine unscharfe Innenkontur, bei direkter Aufsicht auch als *vollständiges Ringzeichen* bezeichnet (Abb. 1d).

Rechtsseitige Divertikel treten im Gegensatz zu linksseitigen Divertikeln meist solitär auf, sind histopathologisch echte transmurale Divertikel und betreffen vergleichsweise jüngere PatientInnen. Sie führen jedoch zu keiner erhöhten Rezidivrate nach akuter Divertikulitis und konservativer Therapie [20]. Kolonische Riesendivertikel, definiert als Divertikel > 4 cm, sind eine sehr seltene Manifestation und v. a. im Colon sigmoideum zu finden. Bei einer Divertikulose finden sich multiple Divertikel, meist im Kolon sigmoideum, die zu einer moderaten Wandverdickung, einer luminalen Engerstellung sowie einer tiefen Haustrierung führen (Abb. 2a).

## Entzündliche Pathologien des Kolons

Neben polypoiden und neoplastischen Pathologien kann bildgebend eine Reihe weiterer gutartiger Erkrankungen festgestellt werden, die das Kolonlumen betreffen. Die Prognose einer akuten Kolitis hängt vom zeitgerechten Beginn einer zielgerichteten Therapie ab. Die Ätiologie kann allerdings aufgrund der unspezifischen Erscheinungsformen klinisch schwer zu bestimmen sein. Daher ist es wichtig, das topografische Erscheinungsbild und die spezifisch radiologischen Befundkriterien der verschiedenen Erkrankungen zu kennen und im klinischen Kontext gemeinsam mit den Laborwerten und Stuhlproben richtig zu beurteilen [21].

### Divertikulitis

Die akute Divertikulitis stellt mit knapp 4 % eine häufige Diagnose bei PatientInnen mit akuten Bauchschmerzen in der Notfallaufnahme dar. 10–25 % der PatientInnen mit einer Divertikulose werden in ihrem Leben an einer Divertikulitis erkranken [22]. Die CT weist die höchste Sensitivität und Spezifität für die Diagnose einer Divertikulitis auf [23]. Verschiedene CT-Scores wie der Hinchey-, der Siewert- und der mNeff-Score sowie die Klassifikation nach der World Society of Emergency Surgery (WSES), wurden entwickelt, um die Erkrankung zu graduieren und den klinischen Verlauf abzuschätzen. Die WSES-Klassifikation [24] unterscheidet bei der akuten Erkrankung zwischen der unkomplizierten, auf die Kolonwand beschränkten Divertikulitis mit perikolischer Entzündungsreaktion und der komplizierten Divertikulitis mit Mikroabszessen und freier Flüssigkeit (Tab. 1). Eine CT-gesteuerte, interventionelle Abszessdrainage kann den klinischen Verlauf der PatientInnen vor einem chirurgischen Eingriff verbessern. Nach Abklingen der Akutphase zeigen das Ausmaß der Wandverdickung und geringer auch die residuale Lumenweite bei der CT-Kolonographie einen direkten Zusammenhang mit dem Outcome der PatientInnen [25].

**Table 1:** 

*Unkomplizierte Divertikulitis*
Verdickte Darmwand, perikolische Entzündung
*Komplizierte Divertikulitis*
1A: Mikroperforation mit perikolischen Gaseinschlüssen oder geringer Flüssigkeitsansammlung ohne Abszessformation (< 5 cm von entzündetem Segment)
1B: mit Abszessformation ≦ 4 cm
2A: mit Abszessformation > 4 cm
2B: perikolische Gaseinschlüsse > 5 cm von betroffenem Segment entfernt
3: diffuse Flüssigkeit in zwei Quadranten ohne freie Gaseinschlüsse
4: diffuse Flüssigkeit mit entfernten Gaseinschlüssen

Die radiologische Differenzierung einer chronischen Divertikulitis von einem Kolonkarzinom kann mitunter anspruchsvoll sein. Die größere Länge des betroffenen Segments (> 10 cm), eine geringere Wandverbreiterung (< 2 cm), fehlende Schulterbildung im Randbereich, eine erhaltene Haustrierung, nachweisbare Divertikel im betroffenen Segment und eine fehlende perikolische Lymphadenopathie sprechen für eine chronische Entzündung.

### Infektiöse Kolitis

Eine Entzündung des Dickdarms kann durch bakterielle, virale, pilzartige oder parasitäre Keime verursacht werden. Meist sind diese Erkrankungen selbstlimitierend und eine spezielle Bildgebung ist für die Diagnose nicht erforderlich. Eine CT kann allerdings notwendig sein, um das Ausmaß der Erkrankung bzw. eventuelle Komplikationen zu diagnostizieren. Typische radiologische Zeichen einer Kolitis sind eine diffuse Verdickung der Kolonwand, oftmals mit einer deutlich erkennbaren Wandschichtung, dem „target sign“. Es entsteht durch ein submukosales Ödem und eine vermehrte mukosale Kontrastmittelaufnahme (Abb. 4b, c). Flüssigkeitsspiegel insbesondere im linksseitigen Kolon und extrakolonische Pathologien wie ödematöse Fettgewebsveränderungen, eine verstärkte Vaskularisation, eine Lymphadenopathie und freie Flüssigkeit bestärken die Diagnose. Luminale Inhalte fehlen jedoch oft in den betroffenen Segmenten („empty colon sign“).

**Figure 3:**
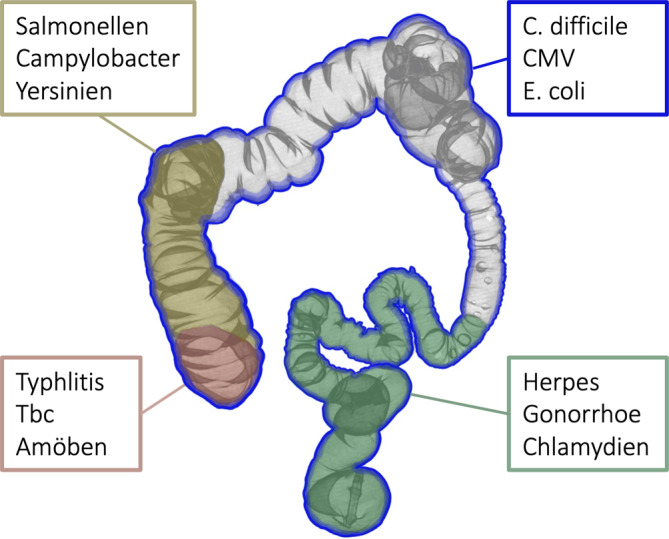

Die geographische Verteilung der Veränderungen im Kolon weist auf mögliche pathogene Erreger hin (Abb. 3).

**Figure 4:**
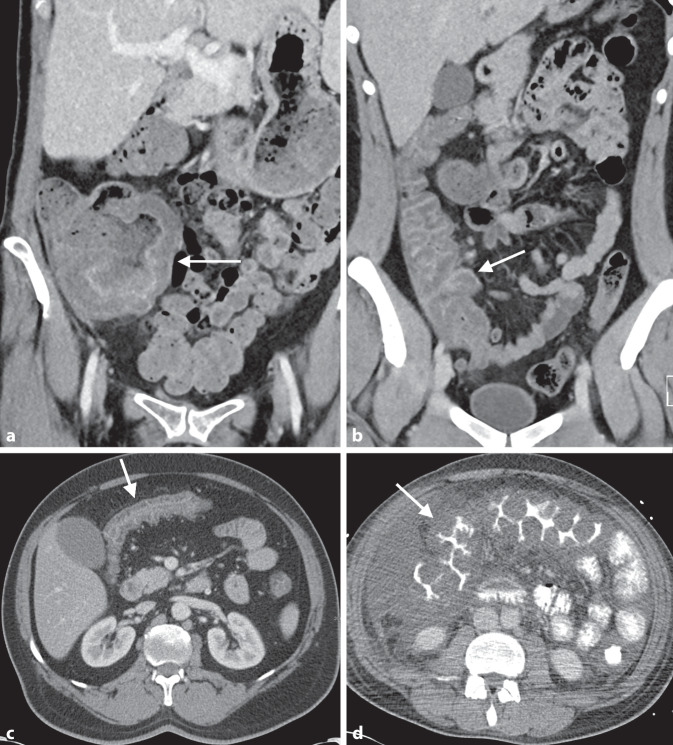

Die Typhlitis ist eine vor allem das Zökum und terminale Ileum betreffende Entzündung immunsupprimierter PatientInnen. Aufgrund der hohen Mortalität handelt es sich um einen medizinischen Notfall (Abb. 4a).

Eine Sonderform der infektiösen Enterokolitiden ist die Infektion mit *Clostridioides difficile*, die oft sekundär nach Antibiotikagabe auftritt. Typisch bei dieser Erkrankung ist bei einer ausgeprägten Darmwandverdickung das „thumbprinting sign“, das durch stark verdickte ödematöse Haustren entsteht und durch die Füllungsdefekte Impressionen durch einen Daumen simuliert, sowie das Akkordeon-Zeichen, das durch positives orales Kontrastmittel oder verstärkt vaskularisierte Mukosa zwischen den verdickten Haustren entsteht (Abb. 4d).

### Chronisch-entzündliche Darmerkrankungen

Ungefähr ein Drittel der PatientInnen mit Morbus Crohn hat eine auf das Kolon beschränkte Erkrankung, von denen ca. 20 % innerhalb des Krankheitsverlaufes einen Dünndarmbefall erleben [26]. Darmwandverdickungen sind bei dieser Patientengruppe typischerweise stärker ausgeprägt mit einer mittleren Wanddicke von 11–13 mm bei M. Crohn im Vergleich zu 7,8 mm bei Colitis ulcerosa [27]. Die exzentrisch segmentalen Veränderungen bei M. Crohn werden oft von einer Proliferation des mesenterialen Fettgewebes begleitet (Tab. 2) und betreffen häufiger das rechtsseitige Kolon. Penetrierende Veränderungen wie Sinustrakte, enterokolische oder kolovesikale Fisteln, entzündliche Pseudotumoren oder Abszesse sind typische Komplikationen dieser transmuralen Erkrankung.

**Table 2:** 

Crohn Kolitis	Colitis ulcerosa
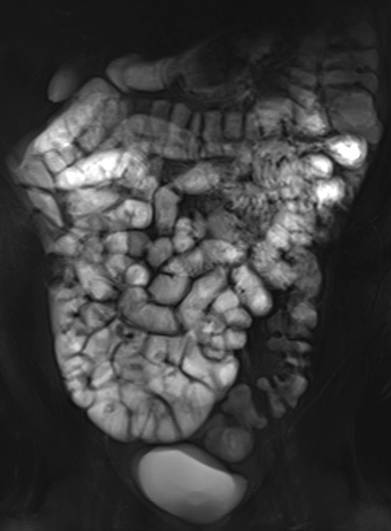 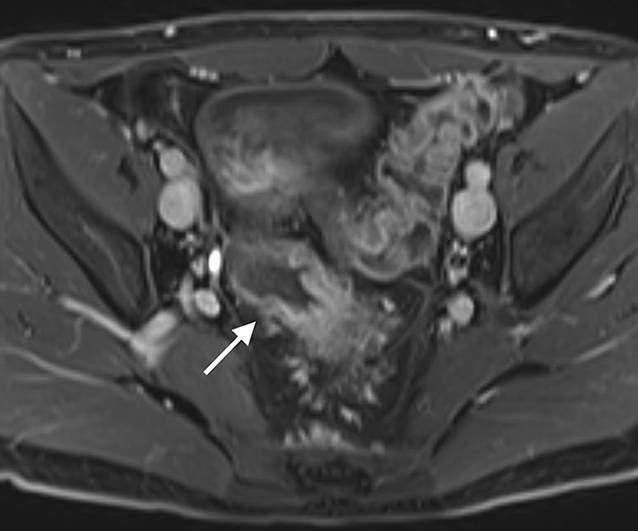	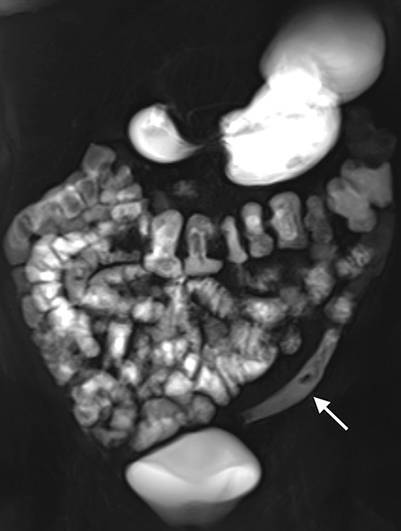 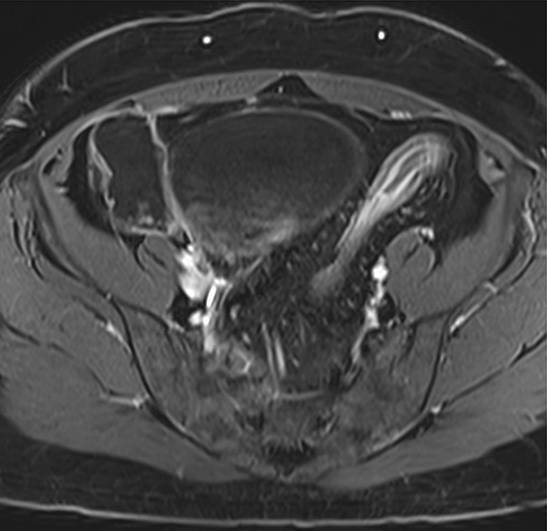
– diskontinuierlich („skip lesions“, *Pfeil*) – deutliche Darmwandverdickung – transmural – fistulierende Veränderungen – +terminales Ileum?	– kontinuierlich – kontinuierlich Wandverdickung <1cm – mukosal und submukosal – Dehaustrierung („Gartenschlauch“, *Pfeil*) – ausgehend vom Rektum

Aktive Entzündungszeichen in der MR-Enterographie sind der Nachweis kolonischer Ulzera, ein verstärkt mukosales Ödem sowie ein murales und perikolisches Ödem auf T2-gewichteten fettsupprimierten Sequenzen, das Ausmaß einer Verbreiterung der Darmwand und eine Diffusionsrestriktion. Die Unterscheidung zwischen aktiv-entzündlichen und fibrotischen Anteilen, die oftmals in Kombination auftreten, ist allerdings weiterhin schwierig. Neue bildgebende Methoden könnten zukünftig dabei behilflich sein [28].

Im Gegensatz zu einer Crohn-Kolitis sind die Veränderungen bei der Colitis ulcerosa typischerweise kontinuierlich und symmetrisch mit distaler Betonung. Mit Ausnahme der Backwash-Ileitis ist das terminale Ileum nicht betroffen. Zu Beginn der Erkrankung können die Haustren ödematös und verdickt sein, während bei einem chronischen Verlauf ein Haustrenverlust mit einer Verkürzung und einer gartenschlauchartigen Konfiguration des Kolons beobachtet werden kann (Tab. 2).

Die Differenzierung zwischen infektiöser und chronisch-entzündlicher Kolitis kann radiologisch schwierig sein. Eine Vergleichsstudie von Plastaras et al. hat als typische Zeichen der infektiösen Kolitis das „empty colon sign“, das kontinuierliche Befallsmuster und das Fehlen vergrößerter Lymphknoten beschrieben, wohingegen die verstärkte mesenteriale Gefäßzeichnung („comb sign“), die Mitbeteiligung des Dünndarms und vergrößerte Lymphknoten für eine chronisch-entzündliche Genese sprechen [29].

### Ischämische Kolitis

Im Ruhezustand erhält der menschliche Darm ca. 20 % des Blutvolumens, von denen zwei Drittel die intestinale Mukosa versorgen, wohingegen postprandial durch die splanchnische Autoregulation die Versorgung auf 35 % gesteigert werden kann [30]. Die Ischämie des Kolons stellt die häufigste Form einer gastrointestinalen Ischämie dar mit einer in den letzten Jahrzehnten ansteigenden Inzidenzrate von ca. 22,9 Fällen je 100.000 Personenjahren [31].

Kollateralsysteme zwischen der A. colica media und der A. colica sinistra wie die Drummond-Anastomose zwischen darmwandnahen Ästen und die inkonstant vorhandene Riolan-Anastomose im Mesokolon sichern eine Blutversorgung des gesamten Kolons. Die Wasserscheiden-Grenzzonen sind daher besonders anfällig für nichtokklusive ischämische Folgeschäden: die linke Flexur (Griffith-Punkt) durch schwach ausgebildete Kollateralen sowie das Rektosigmoid (Sudeck-Punkt) distal der sigmoidalen Kollateralen.

In der CT finden sich bei akuter arterieller Minderdurchblutung eine papierdünne Wand und perikolische Verdichtungen, oft in segmentaler Ausdehnung. Zusätzlich ist auf ein fehlendes Enhancement der Wand, auf eine Darmdilatation als Zeichen einer ischämischen Schädigung des Plexus myentericus und auf eine Pneumatosis coli zu achten (Abb. 6a). Das Vorliegen einer Pneumatose alleine sollte allerdings nicht als Zeichen einer irreversiblen Nekrose gewertet werden, da besonders bei einer nichtokklusiven mesenterialen Ischämie (NOMI) die Pneumatose früher auftreten und zu einer Überdiagnose einer transmuralen Nekrose führen kann. Andererseits sollte bei einer okklusiven Ischämie eine Darmdilatation als frühes und häufigeres Zeichen einer relevanten Minderperfusion berücksichtigt werden [32]. PatientInnen mit einer rechtsseitigen ischämischen Kolitis und freier Flüssigkeit haben oft einen schwereren Krankheitsverlauf mit Notwendigkeit eines chirurgischen Eingriffs [33].

**Figure 5:**
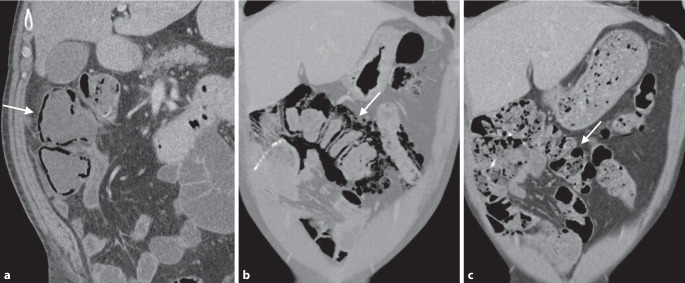

Mit der Reperfusion können abhängig von der Dauer der Ischämie verschiedene Grade der Darmwandschädigung erkennbar werden und durch die venöse Gefäßstauung zu einer deutlichen, oft hyperdensen Darmwandverdickung mit einer Dilatation der mesenterialen Venen führen. In der chronischen Phase können durch Fibrose und Haustrenverlust ischämisch bedingte kolonische Strikturen entstehen [34].

Eine benigne Form einer Pneumatosis coli, auch als zystoide Form bezeichnet, kann u. a. beobachtet werden bei PatientInnen mit pulmonalen Erkrankungen wie einer chronisch-obstruktiven Lungenerkrankung (COPD), aber auch bei einer mechanischen Schädigung der Kolonmukosa oder einer erhöhten mukosalen Permeabilität im Rahmen einer Infektion, Inflammation, nach Organtransplantationen oder unter einer Kortikosteroidtherapie (Abb. 5b, c). Neben unterschiedlichen klinischen und laborchemischen Befunden weisen das Fehlen einer Darmwandverdickung, perikolisch entzündlicher Veränderungen, einer pathologischen Darmdistension, eines Pneumoportogramms sowie eine reguläre Kontrastmittelaufnahme der Darmwand auf eine benigne Form hin [35]. Gerade im Kolon ist darauf zu achten, dass Stuhlinhalt vermischt mit Gas oder Gaseinschlüsse zwischen Haustren eine Pneumatose imitieren können.

### Medikamenteninduzierte Kolitis

Der vermehrte Einsatz medikamentöser Therapien wie Immunologika, Biologika oder Chemotherapeutika hat zu einer Zunahme gastrointestinaler Nebenwirkungen geführt. Die computertomographischen Zeichen einer medikamenteninduzierten Kolitis entsprechen denen infektiöser oder anderer nichtinfektiöser Kolitiden, eine Diagnose ist mithilfe der Medikamentenanamnese zu stellen.

**Figure 6:**
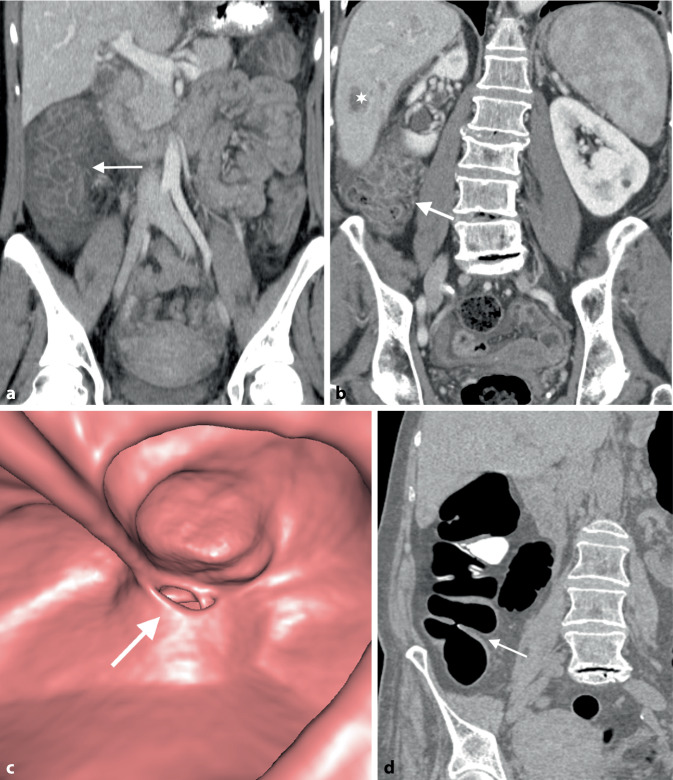

Eine seltene, jedoch spezifische Komplikation nach chronischer Einnahme nichtsteroidaler Antirheumatika ist die NSAR(nichtsteroidale Antirheumatika)-Kolopathie mit Entwicklung schmaler webartiger Stenosen im Kolon (Abb. 6c, d; [36]).

### Andere Entzündungen des Kolons

Eine Strahlenkolitis wird vor allem nach Radiotherapie des Beckens im Rektosigmoid beobachtet. Die Graft-versus-host-Erkrankung (GVHD) ist eine Autoimmunerkrankung nach allogener Stammzelltransplantation und zeigt sich meist als Ileokolitis mit mukosaler Hyperämie, einem Halo-Zeichen als Ausdruck des submukosalen Ödems und perikolisch ödematösen Veränderungen. Die seltene eosinophile Kolitis bei PatientInnen mit allergischer Genese ist mit einer laborchemischen Eosinophilie kombiniert (Abb. 6a).

## Andere Pathologien des Kolons

Eine wichtige Differenzialdiagnose zu entzündlichen Dickdarmerkrankungen ist die *portale Hypertensionskolitis*. Die Prävalenz in Zirrhotikern variiert zwischen 25 und 70 %. Die meist asymptomatische Veränderung kann durch chronische Blutungen mit Eisenmangelanämie klinisch manifest werden [37]. Computertomographisch ist eine symmetrisch ödematös verdickte Darmwand vor allem des rechtsseitigen Kolons auffällig mit verstärktem Enhancement der Mukosa als Zeichen der ursächlichen chronisch-venösen Stauung im Rahmen der portalen Hypertension (Abb. 6b). Dieses radiologische Bild bei gastrointestinal asymptomatischen PatientInnen mit schwerer Zirrhose erfordert daher keine weitere Abklärung oder Intervention [38]. Ein ähnliches radiologisches Bild ist auch bei einer ausgeprägten Herzinsuffizienz zu beobachten.

## Fazit für die Praxis

- Die abdominale CT und MRT gehören zu den nichtinvasiven Standardmethoden in der Diagnostik luminaler kolorektaler Erkrankungen.
- Die CT-Kolonographie ist eine erprobte Alternative zur Koloskopie für die Detektion kolorektaler Polypen und Karzinome.
- Trotz überlappender radiologischer Befunde können charakteristische bildgebende Merkmale richtungsweisend für eine genaue Diagnose sein und dadurch eine zielgerichtete Behandlung ermöglichen.
- Ein längeres Zeitintervall nach oraler Kontrastmittelgabe im Rahmen einer Enterographie kann für eine ausreichende Distension des Kolons sorgen; dies stellt eine Alternative zu einer rektalen Füllung dar.
